# *Salmonella* infection among the pediatric population at a tertiary care children’s hospital in central Nepal: a retrospective study

**DOI:** 10.3389/fmicb.2023.1218864

**Published:** 2023-09-29

**Authors:** Nayanum Pokhrel, Ramhari Chapagain, Chandan Kumar Thakur, Ajaya Basnet, Isha Amatya, Rajan Singh, Raghav Ghimire

**Affiliations:** ^1^Nepal Health Research Council, Kathmandu, Nepal; ^2^Department of Pediatrics, Kanti Children’s Hospital, Kathmandu, Nepal; ^3^Shi-Gan International College of Science and Technology, Kathmandu, Nepal; ^4^Provincial Hospital, Malangwa, Nepal; ^5^Department of Pediatric Cardiology, Shahid Gangalal National Heart Centre, Kathmandu, Nepal

**Keywords:** antibiogram, blood culture, enteric fever, pediatric population, serotypes, salmonella infection

## Abstract

**Background:**

Typhoid fever, an infective bacterial disease, is capable of causing fatal systemic infection in humans, and in an era of antimicrobial resistance, it has become of public health importance. This study aimed to investigate the laboratory diagnosis of *Salmonella* bloodstream infection, its serotype, antimicrobial resistance pattern, and seasonal variation at a tertiary care children’s hospital.

**Methods:**

We undertook a retrospective, cross-sectional study by reviewing hospital-based laboratory records of patients whose blood culture samples were submitted from the outpatient department to the laboratory of a tertiary care children’s hospital in Kathmandu, Nepal, from January 2017 to January 2019.

**Results:**

Among the total blood culture samples obtained (*n* = 39,771), bacterial isolates (*n* = 1,055, 2.65%) belonged either to the Genus *Enterobacteriaceae* or Genus *Acinetobacter*. Altogether (*n* = 91, 8.63%), isolates were positive for *Salmonella* spp., which were further identified as *Salmonella enterica* subsp. *enterica* ser. Typhi (*n* = 79, 7.49%), *Salmonella enterica* subsp. *enterica* ser. Paratyphi A (*n* = 11, 1.04%), and *Salmonella enterica* subsp. *enterica* ser. Paratyphi B (*n* = 1, 0.1%). The median age of patients was 6  years (IQR: 4–9), with male and female patients constituting (*n* = 53, 58.24%; OR, 1.0; 95% CI, 0.60–1.67) and (*n* = 38, 41.76%; OR, 0.98; 95% CI, 0.49–2.05) cases, respectively. The disease was observed throughout the year, with a high prevalence toward the spring season (March–May). An antibiogram showed resistance more toward nalidixic acid with *S.* Typhi, comprising half the isolates (*n* = 52, 65.82%; *p* = 0.11). Resistance toward β-lactams with β-lactamase inhibitors (amoxicillin/clavulanate; 1.27%) was seen in a single isolate of *S.* Typhi. The multidrug resistance pattern was not pronounced. The multiple antibiotic resistance (MAR) index was in the range between 0.14 and 0.22 in *S.* Typhi and 0.22 and 0.23 in *S.* Paratyphi.

**Conclusion:**

*Salmonella* Typhi was the predominant ser. Infection was common among children between 1 and 5 years of age, showing male predominance and with the spring season contributing to a fairly higher number of cases. Antimicrobial susceptibility testing of *S.* Typhi showed more resistance toward nalidixic acid, with only a single isolate resistant to β-lactamase inhibitors (amoxicillin/clavulanate). Alarming multidrug resistance patterns were not observed. The MAR index in this study indicates the importance of the judicious use of antimicrobials and hospital infection prevention and control practices.

## 1. Introduction

Typhoid fever is a life-threatening systemic infection affecting 11–20 million people and with almost 128,000 to 161,000 people dying from it each year in numerous growing parts of the WHO African, Eastern Mediterranean, South-East Asia, and Western Pacific Regions (WHO, 2018). This disease is associated with the bacterium *Salmonella enterica* serotype Typhi, a member of the family *Enterobacteriaceae*, which shows seropositivity for a range of both capsular and flagellar antigens that include lipopolysaccharide antigens O9 and O12, protein flagellar antigen Hd, and polysaccharide capsular antigen Vi (Parry et al., 2002). Typhoid fever is a disease of significant importance in overcrowded and unsanitary conditions, as seen in many developing countries and, infrequently, in developed nations, where only sporadic cases are reported among travelers returning from endemic areas (Osler, 1912; Ackers et al., 2000). Several factors come into play for the transmission of this infection, some of which include food consumption from street vendors such as ice cream or flavored iced drinks (Black et al., 1985; Luby et al., 1998), substandard housing conditions, and personal hygiene, as well as the recent use of antimicrobial drugs (Luby et al., 1998).

The symptoms of the disease include protracted fever, lassitude, headache, nausea, abdominal pain, constipation or diarrhea, and an occasional rash, with death in severe and complicated cases. Chloramphenicol, once regarded as a game changer in the management of severe, incapacitating, and frequently lethal disease into a treatable illness, saw a shift in its treatment guidelines, including management with the fluoroquinolone group of drugs, paving the path for newer-generation cephalosporins and azithromycin in affected areas (Woodward et al., 2004; WHO, 2014).

Nonetheless, among the preventive measures aimed at reducing the risk of typhoid fever, vaccination also serves as a good strategy for *S.* Typhi prevention.

The first identification of typhoid infection in Nepal was in a British-Nepalese soldier in 1984, followed by an infant in 1989 (Klonin et al., 1989). Since then, the disease has been reported in all regions of Nepal and has affected individuals of all ages, with a disproportionately high number of cases occurring among children and young adults (Gupta et al., 2021). Moreover, the growing concern about the rise of antimicrobial resistance in all infectious diseases has made *S.* Typhi an important bacterium of concern for treatment in endemic regions such as Nepal. Therefore, the primary objective of this study was to determine the presence of typhoidal *Salmonella* in the blood culture of pediatric patients attending the outpatient department of a tertiary care children’s hospital. The study also aimed to identify the serotype of *Salmonella*, analyze its antimicrobial resistance pattern, and assess any seasonal variations over 2 years.

## 2. Methods

### 2.1. Study design and patients

A retrospective, cross-sectional study was conducted by retrieving documented paper-based hospital laboratory records from the Department of Microbiology at Kanti Children’s Hospital, Kathmandu, Nepal. The main catchment area for the hospital is the Kathmandu Valley (population ~3,025,386; Central Bureau of Statistics, 2021), but as the only government-run children’s hospital in Nepal, it also caters to children from all other regions of the country. Blood culture samples collected from pediatric patients visiting the outpatient department of the hospital were reviewed. The demographic data of the patients, isolated organisms, and their respective antibiograms within 2 years duration (January 2017 to January 2019) were included. Any incomplete data were excluded from the study. The study was approved by the Institutional Review Committee (IRC) of Kanti Children’s Hospital, Maharajgunj, Kathmandu, Nepal (IRC No: 969). Laboratory tests were performed as a part of routine diagnostic procedures based on the clinician’s need. Hence, patient-informed consent was not applicable.

### 2.2. Laboratory procedure

Approximately 3 mL of blood sample was inoculated in Bactalert culture bottles. Once growth was indicated by the Bactalert system, biochemical tests using commercially available media preparations (Hi-Media Laboratories, Mumbai, India) were prepared in-house using standard methods and techniques (Collee et al., 1996) for bacterial identification. A slide agglutination test using commercially available antisera was performed for *Salmonella* spp. following the manufacturer’s instruction (Denka Seiken Co., Ltd., Chuo-Ku, Tokyo, Japan), and the Genus *Salmonella* and its serogroups were identified using the antigenic classification of the Kauffmann–White Scheme. An antimicrobial susceptibility test by the Kirby–Bauer disc diffusion method was performed using a Muller Hinton agar (Hi-Media Laboratories, Mumbai, India) following the 29th edition CLSI guidelines (Weinstein et al., 2019). Antibiotic discs included in this study were Ampicillin (10 μg), Amoxicillin (10 μg), Cefixime (5 μg), Cefotaxime (30 μg), Cefpodoxime (30 μg), Ceftriaxone (30 μg), Ceftazidime (30 μg), Cefepime (30 μg), Ciprofloxacin (5 μg), Ofloxacin (5 μg), Trimethoprim/sulfamethoxazole (Cotrimoxazole; 1.25/23.75 μg), Chloramphenicol (30 μg), Nalidixic acid (30 μg), Amoxicillin/clavulanate (20/10 μg), Ampicillin/sulbactam (10/10 μg), and Imipenem (10 μg; Hi-Media Laboratories, Mumbai, India). An antimicrobial susceptibility pattern was determined as sensitive, intermediate, and resistant according to the 29th edition CLSI guidelines (Weinstein et al., 2019). Furthermore, the multiple antibiotic resistance (MAR) index was calculated as the ratio of the number of resistant antibiotics to which the organism is resistant to the total number of antibiotics the organism is exposed to. MAR index values more than 0.2 specify high-risk sources of contamination where antibiotics are frequently used (Krumperman, 1983).

### 2.3. Sample workflow

See Figure 1.

**Figure 1:**
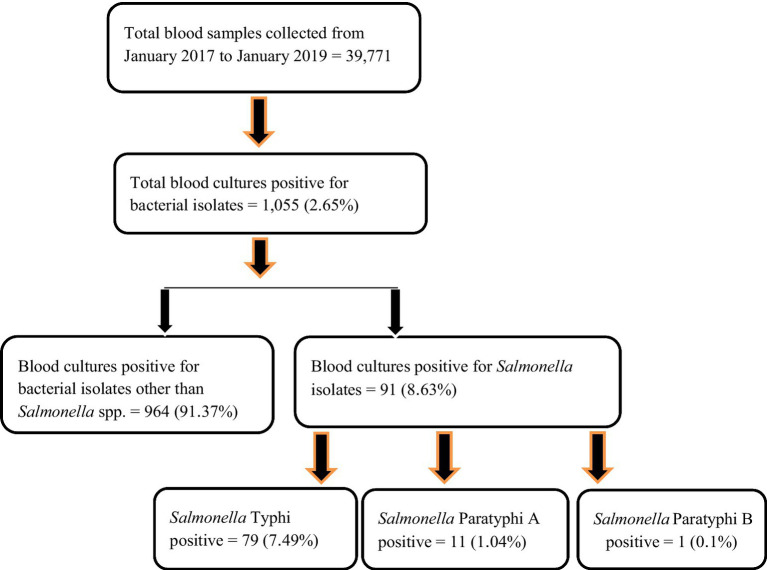
Sample workflow of *Salmonella* isolates from blood samples of pediatric patients’ (*n* = 91).

### 2.4. Statistical analysis

All the data were entered into a Microsoft Excel 2007 spreadsheet from the paper-based hospital records and further analyzed by SPSS software version 17. All the descriptive and inferential data were calculated using SPSS software. The Chi-square test was used to analyze the categorical data.

## 3. Results

**Figure 2:**
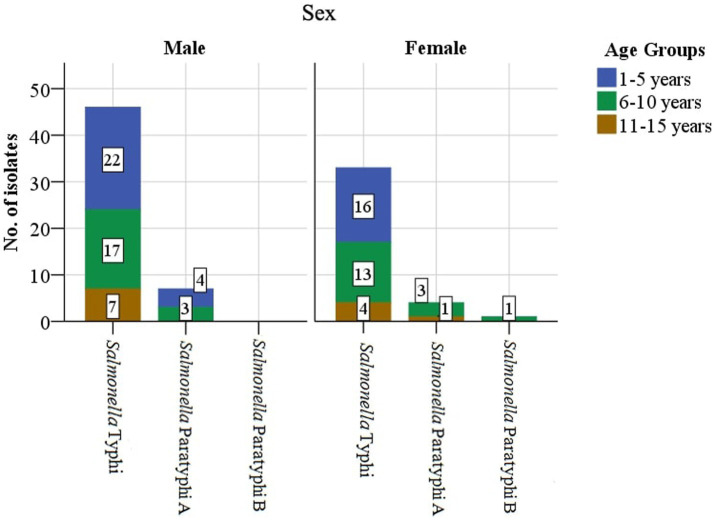
Isolation of *Salmonella* spp. based on the patients’ demographics (*n* = 91).

**Figure 3:**
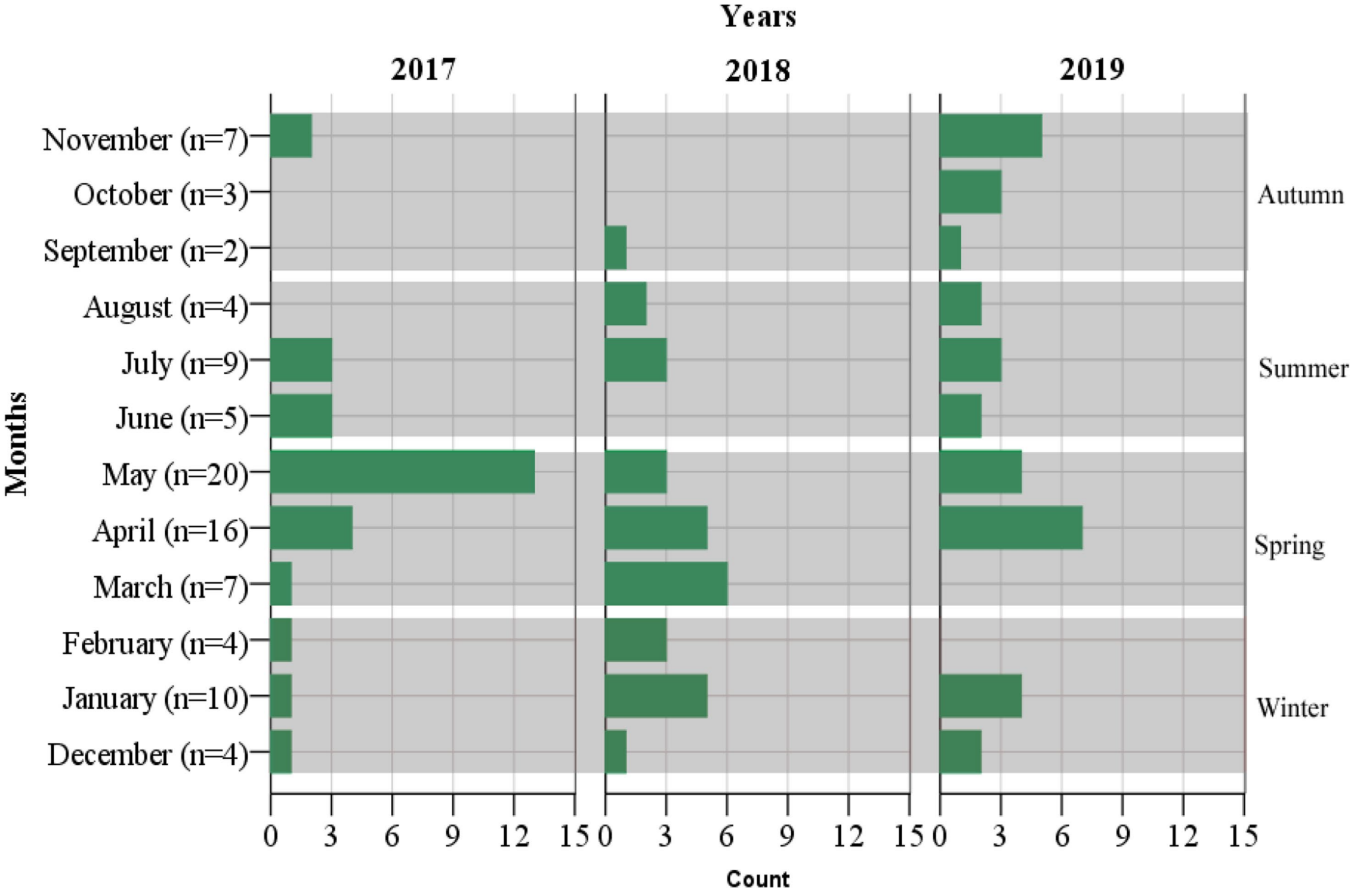
Incidence of enteric fever based on months, seasons, and years (*n* = 91).

Enteric fever is common in Nepal, and Kathmandu Valley is an endemic region for this infection due to its known risk of substandard water quality at the community level and its poor sanitation and hygiene, jeopardizing the health of individuals and increasing their chances of acquiring infection (Karkey et al., 2013). Our hospital is the only tertiary-level, government-run children’s hospital, located in Maharajgunj, Kathmandu, and it caters to hundreds of children from different socio-economic statuses coming to receive quality treatment. Among the total blood culture samples (*n* = 39,771) collected, (*n* = 1,055, 2.65%) samples tested positive for bacteria belonging either to the Genus *Enterobacteriaceae* or Genus *Acinetobacter*. The culture-positive isolates (*n* = 1,055, 2.65%) were further distributed into two groups. The first group constituted of the Genus *Enterobacteriaceae* (excluding *Salmonella* spp.) and Genus *Acinetobacter* (*n* = 964, 91.37%), with the second group constituting of the Genus *Enterobacteriaceae* comprising exclusively of *Salmonella* spp. (*n* = 91, 8.63%). The *Salmonella* spp. (*n* = 91, 8.63%) were further identified as *Salmonella* ser. Typhi (*n* = 79, 7.49%), Paratyphi A (*n* = 11, 1.04%), and Paratyphi B (*n* = 1, 0.1%). The affected age group was 1–5 years of age, followed by 6–10 years, with the least being 11–15 years. The median age of the patients was 6 years, with an interquartile range of (IQR: 4–9) years (Figure 2). Sex-wise distribution showed the prevalence of *Salmonella* infection among both male (*n* = 53, 58.24%; OR, 1.0; 95% CI, 0.60–1.67), and female patients (*n* = 38, 41.76%; OR, 0.98; 95% CI, 0.49–2.05; Figure 2). Between 2017 and 2019, the highest number of cases was seen during the spring season (March–May; OR, 1.84; 95% CI, 0.46–7.33) and the least during autumn (September to November; OR, 0.51; 95% CI, 0.60–4.30; Figure 3).

Among the tested antimicrobials, resistance was observed in quinolones and their derivatives (nalidixic acid), showing (*n* = 60, 88.23%) resistance to all the *Salmonella* isolates, followed by fluoroquinolone resistance (ciprofloxacin; *n* = 29, 34.11%) and ofloxacin (*n* = 11, 13.92%). Moreover, the penicillin group of antimicrobials was second in line with the overall resistance pattern observed, which was more among amoxicillin (*n* = 7, 24.13%) and ampicillin (*n* = 5, 10.41%). Among the combination drugs, β-lactams with β-lactamase inhibitors (amoxicillin/clavulanate) showed (*n* = 1, 20%) resistant isolate (Table 1). Among the total *Salmonella* ser.isolated, ser. Typhi (*n* = 79, 86.81%) exhibited (*n* = 52, 65.82%) resistance to quinolones and their derivatives (nalidixic acid) and resistance to fluoroquinolones such as ciprofloxacin (*n* = 24, 30.38%). Similarly, the bacterium also showed resistance toward the penicillin group of drugs, such as amoxicillin (*n* = 6, 7.59%) and ampicillin (*n* = 3, 3.8%). Furthermore, β-lactams with β-lactamase inhibitors had a single isolate of *S.* Typhi resistant to amoxicillin/clavulanate (*n* = 1, 1.27%; Table 1). The median multiple antibiotic resistance index or the MAR index values for *S.* Typhi were in the range between 0.14 and 0.22. Likewise, for *S.* Paratyphi, 0.22–0.23 was the mean MAR index (Table 2). There were no clinical outcomes in the form of hospital admission for any of the known complications of typhoid fever among the outpatients visiting the hospital, who were clinically diagnosed with enteric fever in the 2-year study duration.

**Table 1:** Antimicrobial susceptibility pattern among the isolates of *Salmonella* spp. (*n* = 91).

Antibiotics			*Salmonella* spp.					
			*S.* Paratyphi A (*n* = 11)	*p-*value	*S.* Paratyphi B (*n* = 1)	*p-*value	*S.* Typhi (*n* = 79)	*p-*value
Penicillins	Ampicillin	Resistant (*n* = 5)	2 (18.18%)	0.15	0 (0%)		3 (3.80%)	0.15
		Intermediate (*n* = 3)	1 (9.09%)		0 (0%)		2 (2.53%)	
		Susceptible (*n* = 40)	4 (36.36%)		0 (0%)		36 (45.57%)	
	Amoxicillin	Resistant (*n* = 7)	1 (9.09%)	1.00	0 (0%)		6 (7.59%)	1.00
		Susceptible (*n* = 22)	2 (18.18%)		1 (100%)		19 (24.05%)	
β-lactams with β-lactamase inhibitors	Amoxicillin/clavulanate	Resistant (*n* = 1)	0 (0%)		0 (0%)		1 (1.27%)	-*
		Susceptible (*n* = 4)	0 (0%)		0 (0%)		4 (5.06%)	
	Ampicillin/sulbactam	Susceptible (*n* = 3)	0 (0%)		0 (0%)		3 (3.80%)	
Cephalosporins	Cefotaxime	Susceptible (*n* = 11)	1 (9.09%)		0 (0%)		10 (12.66%)	
	Ceftriaxone	Resistant (*n* = 1)	0 (0%)		0 (0%)		1 (1.27%)	1.00
		Susceptible (*n* = 57)	7 (63.63%)		0 (0%)		50 (63.29%)	
	Cefpodoxime	Intermediate (*n* = 1)	0 (0%)		0 (0%)		1 (1.27%)	
		Susceptible (*n* = 5)	0 (0%)		0 (0%)		5 (6.33%)	
	Cefexime	Resistant (*n* = 1)	0 (0%)		0 (0%)		1 (1.27%)	1.00
		Susceptible (*n* = 79)	11 (100%)		0 (0%)		68 (86.08%)	
	Ceftazidime	Susceptible (*n* = 16)	1 (9.09%)		0 (0%)		15 (18.99%)	
	Cefepime	Susceptible (*n* = 10)	0 (0%)		1 (100%)		9 (11.39%)	
Aminoglycosides	Amikacin	Resistant (*n* = 10)	1 (9.09%)	-*	0 (0%)		9 (11.39%)	-*
	Gentamicin	Resistant (*n* = 2)	0 (0%)		1 (100%)	-*	1 (1.27%)	-*
Quinolones and their derivatives	Nalidixic acid	Resistant (*n* = 60)	7 (63.63%)	0.09	1 (100%)	1.00	52 (65.82%)	0.11
		Susceptible (*n* = 8)	3 (27.27%)		0 (0%)		5 (6.33%)	
	Ofloxacin	Resistant (*n* = 11)	0 (0%)		0 (0%)		11 (13.92%)	0.34
		Intermediate (*n* = 2)	1 (9.09%)		0 (0%)		1 (1.27%)	
		Susceptible (*n* = 66)	9 (81.81%)		0 (0%)		57 (72.15%)	
	Ciprofloxacin	Resistant (*n* = 29)	5 (45.45%)	0.50	0 (0%)		24 (30.38%)	0.53
		Intermediate (*n* = 1)	0 (0%)		0 (0%)		1 (1.27%)	
		Susceptible (*n* = 55)	6 (54.55%)		1 (100%)		48 (60.76%)	
Trimethoprim/sulfamethoxazole		Resistant (*n* = 4)	0 (0%)		0 (0%)		4 (5.06%)	1.00
		Susceptible (*n* = 80)	11 (100%)		1 (100%)		68 (86.08%)	
Chloramphenicol		Resistant (*n* = 3)	0 (0%)		0 (0%)		3 (3.80%)	1.00
		Susceptible (*n* = 22)	2 (18.18)		1 (100%)		19 (24.05%)	

-*, Data not applicable.

**Table 2:** Median multiple antibiotic resistance index among the *Salmonella* spp. (*n* = 91).

S.N.	*Salmonella* spp.	Strain-overall	Strain-specific
1.	*S.* Typhi	0.22	0.14
2.	*S.* Paratyphi	0.23	0.22

## 4. Discussion

Enteric fever is considered one of the leading causes of febrile bacterial illness among adults and children in both developing and developed nations (Sánchez-Vargas et al., 2011). The genetic constitution of *Salmonella* spp. enhances their adaptability in both mammalian and non-mammalian hosts, including non-animated reservoirs, thereby challenging their eradication by conventional methods. In an era of antimicrobial resistance, *Salmonella* strains face a similar fate to any other microorganisms exhibiting resistance to multiple drugs, making treatment an uphill task (Sánchez-Vargas et al., 2011). Our study found that *Salmonella enterica* ser. Typhi is the most common isolate (*n* = 79, 7.49%), followed by *Salmonella enterica* ser. Paratyphi A (*n* = 11, 1.04%) and *Salmonella enterica* ser. Paratyphi B in a single (*n* = 1, 0.1%) case. Historically, a high proportion of *Salmonella* Typhi infections have been reported in Nepal, with a relatively lower proportion of *Salmonella* Paratyphi (Karkey et al., 2010; Shrestha et al., 2014; Thompson et al., 2017), but the trends have been changing over the past two decades, with an increase in *Salmonella* Paratyphi A in some parts of Asia (Karkey et al., 2010; Zellweger et al., 2017).

Among the *Salmonella* isolates, a lower proportion (*n* = 79, 7.49%) of ser. Typhi in our study was comparable to those conducted in various parts of Nepal, contributing to 5.1% and 5.4% of cases (Khanal et al., 2007; Pokharel et al., 2009), but was in contrast (higher, 12.3%, 55.7%, 77.7,% and 85%) to studies published in Pakistan, Nepal, and India, along with population-based surveillance, respectively (Siddiqui et al., 2006; Petersiel et al., 2018; Biswas et al., 2022; Garrett et al., 2022). The findings from our study could be attributed to prior antimicrobial treatment received by the affected age group (1–5 years) before obtaining a blood sample for culture and sensitivity (Britto et al., 2018). Additionally, only 1.04% of Paratyphi A cases according to our estimates are comparable to a publication from Pakistan (Siddiqui et al., 2006) but discordant (higher, 17% and 12%) to few studies from Nepal (Prajapati et al., 2008; Budhathoki et al., 2020) and another population-based enteric fever surveillance (higher, >99%), respectively (Garrett et al., 2022). The variation in Paratyphi A cases in our findings could be attributed to the asymptomatic infections manifested by this ser. (Sood et al., 1999), with relatively young male adults becoming infected (Karkey et al., 2010), which differed from our study population. Only 3% of *Salmonella* ser. Paratyphi B infection cases among the Nepalese population have been reported, rendering it an uncommon bloodstream infection (Pokhrel et al., 2009; Karkey et al., 2010; Zellweger et al., 2017; Garrett et al., 2022), which is quite similar (*n* = 1, 0.1%) to our study observation but differs (higher, 10%) from another scientific publication in Nepal (Budhathoki et al., 2020). The higher proportion of ser. Paratyphi B infection in the latter study reflects the greater predisposition to this infection among older children (11–15 years), who comparatively have a higher exposure to the external environment and outdoor activities than younger ones (1–5 years).

Children in the age group between 1 and 5 years were the most commonly infected (*n* = 42, 46.1%), with the least among those aged between 11 and 15 years (*n* = 12, 13.2%), similarly to the findings in India (Das et al., 2016), Pakistan (Rafiq et al., 2009; Britto et al., 2017), and other studies in the series (Mahle and Levine, 1993; Pang et al., 1995). However, this was in contrast (lower, 26.5%, 21.3%, and 14%,) to other publications in Pakistan, Nepal, and India for the age group between 1 and 5 years, respectively (Siddiqui et al., 2006; Budhathoki et al., 2020; Behera et al., 2021). The age-related variation in our findings highlights the possibility of reduced documentation of the disease, poor clinical suspicion, prior antimicrobial treatment before blood culture, and difficulty in withdrawing blood resulting in poor laboratory and clinical outcomes (Britto et al., 2018), along with immunological reasons such as immature and unstable gut microbiome and gut immune function in children between 1 and 5 years of age, easily exposing them to bacterial infections such as *S*. Typhi in comparison to older ones (Nuriel-Ohayon et al., 2016).

Enteric fever was more common in the male population, constituting more than half (*n* = 53, 58.24%) of the cases, with similar observations made in countries such as India, Nepal, and African countries, as well as in a population-based enteric fever surveillance (Ramaswamy et al., 2010; Rabasa et al., 2012; Singh et al., 2012; Garrett et al., 2022). The variation in sex proportion in our results could be attributed to factors such as prioritizing a male child over a female for treatment in our context and more outdoor activities seen among male children exposing them to the root of infection.

The wet season in Nepal begins from May to November and the dry season from December to February (Sharma et al., 2021). The frequency of typhoid infections in our study is seen throughout the year, but the incidence was high toward the spring season (end of dry season and beginning of wet season) throughout the two-year duration (March 2017 to May 2019). Our findings were concordant with studies conducted in Nepal (Petersiel et al., 2018), India (Ramaswamy et al., 2010), and Vietnam (Lin et al., 2000) but discordant with other studies conducted in Nepal (Karkey et al., 2010), Bangladesh (Dewan et al., 2013), Pakistan (Siddiqui et al., 2006), and Africa (Rabasa et al., 2012), where cases were seen throughout the year with increased frequency during the peak of the wet months (July–October). The isolation of the bacteria throughout the year in our study with comparatively higher prevalence during spring could be subjected to the microbial contamination of drinking water above the recommended levels in Nepal, thereby impacting the health of Nepalese people and specifically children (Farooqui et al., 1991; Parry et al., 2011; UNICEF Nepal, 2018) via various waterborne diseases (Butler et al., 1991; MR and Nair, 2010).

The treatment for enteric fever over the years has become challenging due to multidrug resistance, with the choice of the drug depending on local patterns of antimicrobial resistance, the severity of the disease, availability, and cost of antimicrobials (JA and Mintz, 2010; WHO, 2014). Our results displayed the occurrence of nalidixic acid-resistant *S.* Typhi (*n* = 52, 65.82%; *p* = 0.11), which was low in comparison to other studies in Nepal (Singh et al., 2011; Petersiel et al., 2018) and India (Walia et al., 2006) but high compared to other similar studies within the nation (Singh et al., 2012) and India (Ramaswamy et al., 2010; Bhumbla et al., 2022). Population-based enteric fever surveillance revealed nalidixic acid resistance in 59% of isolates from Pakistan, 57% from India, 44% from Vietnam, and none from Chinese or Indonesian sites in 2008 (Ochiai et al., 2008). Susceptibility to nalidixic acid is thought to be the best interpreter of clinical response to fluoroquinolones (Parry, 2004), and there have been pleas to adjust the fluoroquinolone breakpoints for all *Salmonella* spp. (Aarestrup et al., 2003). The resistance to nalidixic acid in our results indicates reduced susceptibility and poor clinical response to older-generation fluoroquinolones, which is still considered a first-line treatment for enteric fever in Nepal (Maskey et al., 2008), with our study revealing *S.* Typhi (*n* = 24, 30.38%; *p* = 0.53) being resistant to ciprofloxacin. Systematic reviews on antimicrobial resistance in *S.* Typhi conducted worldwide have witnessed 15% resistance to ciprofloxacin, which is lower than our study estimates but analogous to fluoroquinolones resistance observed within the vicinity (Pham et al., 2016). Resistance toward older drugs such as Amoxicillin (*n* = 6, 7.59%; *p* = 1.00), Ampicillin (*n* = 3, 3.80%; *p* = 0.13), Trimethoprim/sulfamethoxazole (*n* = 4, 5.06%; *p* = 1.00), and Chloramphenicol (*n* = 3, 3.80%; *p* = 1.00) was more toward *S.* Typhi, but none of these were statistically significant. Fairly low resistance toward these antimicrobials has been reported in India and Nepal too (Walia et al., 2006; Petersiel et al., 2018). A systematic review on antimicrobial resistance globally among *S.* Typhi reported 25.9%, 37.9%, and 38.8% resistance toward chloramphenicol, cotrimoxazole, and ampicillin and higher resistance (61.2%) toward amoxicillin (Marchello et al., 2020). The lower level of resistance toward chloramphenicol from our findings could also be due to the lower usage of this drug among the pediatric population due to its known adverse events. Evidence showing more sensitivity toward first-line drugs has created a dilemma in the re-usage and recycling concept of the first-line therapy for enteric fever (Pham et al., 2016), with our study results agreeing with this concept. Cephalosporins are the current drug of choice for the treatment of enteric fever in Nepal (Britto et al., 2018), with our laboratory findings showing resistance to only one isolate each of Ceftriaxone (*n* = 1, 1.27%; *p* = 1.00) and Cefixime (*n* = 1, 1.27%; *p* = 1.0) among ser. Typhi, resembling the findings of Prajapati et al. (2008) and Marchello et al. (2020). Minimal resistance was observed in beta-lactam with beta-lactamase inhibitors (amoxicillin/clavulanate; *n* = 1, 1.27%) and was parallel to the results from India (Bhumbla et al., 2022; Biswas et al., 2022) and the findings of a systematic review exhibiting 8.0% resistance toward amoxicillin/clavulanate (Marchello et al., 2020). Among the ser. Paratyphi A isolates, resistance was seen toward Ampicillin (*n* = 2, 18.18%; *p* = 0.13), Amoxicillin (*n* = 1, 9.09%; *p* = 1.00), Nalidixic acid (*n* = 2, 63.63%; *p* = 0.09), and Ciprofloxacin (*n* = 5, 45.45%; *p* = 0.50), with only a single isolate of ser. Paratyphi B resistant only to Nalidixic acid (*n* = 1, 100%; *p* = 1.00). These findings were in line with studies from India and Nepal (Petersiel et al., 2018; Biswas et al., 2022) but were not statistically significant for both the bacteria.

MAR analysis is a risk evaluation tool that differentiates low- and high-risk regions of antibiotic overuse. The MAR index was in the range between 0.14 and 0.22 in *S.* Typhi and 0.22 and 0.23 in *S.* Paratyphi. The MAR index of >0.2 in *S.* Paratyphi and about 0.2 in *S.* Typhi indicates the presence of a high-risk source of contamination from the environment where several antimicrobials are used (Osundiya et al., 2013; Davis and Brown, 2016; Ayandele et al., 2020). The findings from our observations could be attributed to high antibiotic use and high selective pressure in the given environment and insufficient infection prevention and control practices, followed by poor surveillance of antimicrobial susceptibility patterns. Bacterial strains resistant to most classes of antimicrobials are emerging from time to time, hinting at various problems such as the injudicious use of antimicrobials and the lack of rigorous training and workshops on infection prevention and control practices (Osundiya et al., 2013); these need to be acknowledged, and measures to reduce these problems should be addressed. Our data limit the clinical characteristics of the patients, MIC data, and antimicrobial-resistant genes, specifically fluoroquinolones. Moreover, genotyping of the isolates would have enhanced the genetic understanding of the antimicrobials and also compared the lineage drift over the years, specifically in the pediatric population, as reported by other studies within the country.

## 5. Conclusion

In conclusion, this study reveals the prevalence of enteric fever predominantly in children between 1 and 5 years of age, with *S.* Typhi being the most common causative pathogen, the majority of which are nalidixic acid resistant (NARST). Moreover, the multidrug resistance pattern toward *Salmonella* isolates was not apparent, but a comparatively acceptable susceptibility was seen toward the cephalosporin and beta-lactamase inhibitor classification of drugs. Therefore, as far as antimicrobial resistance is concerned, the antimicrobial susceptibility situation does not look alarming. The existence of this bacterium in children raises a general concern regarding hand and food hygiene, along with clean and safe drinking water. It also focuses on the need for public health intervention to raise awareness among children, adults, and food vendors about the disease. The inclusion of typhoid vaccines under the routine immunization program in Nepal for children from 15 months to 15 years of age since 8 April 2022 (UNICEF Nepal, 2022) is a great initiative taken toward controlling the disease, and hopefully, in years to come, we can witness a significantly lesser number of cases.

## Data availability statement

The raw data supporting the conclusions of this article will be made available by the authors, without undue reservation.

## Ethics statement

The studies involving human participants were reviewed and approved by Institutional Review Committee (IRC) of Kanti Children’s Hospital, Maharajgunj, Kathmandu, Nepal (IRC No: 969). Written informed consent from the participants’ legal guardian/next of kin was not required to participate in this study in accordance with the national legislation and the institutional requirements.

## Author contributions

NP: conceptualization, literature search, data curation, and writing—original draft and editing. RC: conceptualization, reviewing, and editing. CT: validation, reviewing, and editing. AB: data analysis, reviewing, and editing. IA: reviewing and editing. RS: reviewing and editing. RG: reviewing and editing. All authors contributed to the article and approved the submitted version.
